# Infrapatellar fat pad-derived mesenchymal stem cell-based spheroids enhance their therapeutic efficacy to reverse synovitis and fat pad fibrosis

**DOI:** 10.1186/s13287-020-02107-6

**Published:** 2021-01-07

**Authors:** Dimitrios Kouroupis, Melissa A. Willman, Thomas M. Best, Lee D. Kaplan, Diego Correa

**Affiliations:** 1 Department of Orthopedics, UHealth Sports Medicine Institute, University of Miami, Miller School of Medicine, 1450 NW 10th Ave (3014), Miami, FL 33136 USA; 2 Diabetes Research Institute & Cell Transplantation Center, University of Miami, Miller School of Medicine, 1450 NW 10th Ave (3014), Miami, FL 33136 USA

**Keywords:** Mesenchymal stem cells (MSC), Infrapatellar fat pad (IFP), CD146 subpopulations, Cell priming, Spheroid cultures, Synovitis, IFP fibrosis, Osteoarthritis

## Abstract

**Background:**

To investigate the in vitro and in vivo anti-inflammatory/anti-fibrotic capacity of IFP-MSC manufactured as 3D spheroids. Our hypothesis is that IFP-MSC do not require prior cell priming to acquire a robust immunomodulatory phenotype in vitro in order to efficiently reverse synovitis and IFP fibrosis, and secondarily delay articular cartilage damage in vivo.

**Methods:**

Human IFP-MSC immunophenotype, tripotentiality, and transcriptional profiles were assessed in 3D settings. Multiplex secretomes were assessed in IFP-MSC spheroids [Crude (non-immunoselected), CD146^+^ or CD146^−^ immunoselected cells] and compared with 2D cultures with and without prior inflammatory/fibrotic cell priming. Functionally, IFP-MSC spheroids were assessed for their immunopotency on human PBMC proliferation and their effect on stimulated synoviocytes with inflammation and fibrotic cues. The anti-inflammatory and anti-fibrotic spheroid properties were further evaluated in vivo in a rat model of acute synovitis/fat pad fibrosis.

**Results:**

Spheroids enhanced IFP-MSC phenotypic, transcriptional, and secretory immunomodulatory profiles compared to 2D cultures. Further, CD146^+^ IFP-MSC spheroids showed enhanced secretory and transcriptional profiles; however, these attributes were not reflected in a superior capacity to suppress activated PBMC. This suggests that 3D culturing settings are sufficient to induce an enhanced immunomodulatory phenotype in both Crude and CD146-immunoselected IFP-MSC. Crude IFP-MSC spheroids modulated the molecular response of synoviocytes previously exposed to inflammatory cues. Therapeutically, IFP-MSC spheroids retained substance P degradation potential in vivo, while effectively inducing resolution of inflammation/fibrosis of the synovium and fat pad. Furthermore, their presence resulted in arrest of articular cartilage degradation in a rat model of progressive synovitis and fat pad fibrosis.

**Conclusions:**

3D spheroids confer IFP-MSC a reproducible and enhanced immunomodulatory effect in vitro and in vivo, circumventing the requirement of non-compliant cell priming or selection before administration and thereby streamlining cell products manufacturing protocols.

## Background

Synovium and infrapatellar fat pad (IFP) tissues have been considered a single anatomical unit [1], actively participating in the modulation of the knee’s intra-capsular homeostasis [2]. As such, this unit serves as a site of immune cell infiltration as well as an active source of multiple pro-inflammatory/pro-fibrotic and articular cartilage catabolic mediators including tumor necrosis factor-alpha (TNF-α), interferon-gamma (IFN-γ), connective tissue growth factor (CTGF), and matrix metalloproteinases (MMPs) [3–9]. Clinically, synovial and IFP inflammation and fibrosis are increasingly recognized with both the onset and progression of joint disease including osteoarthritis (OA) [10–15]. Accordingly, targeting this inflammation and fibrosis could have a potential impact on altering the course of debilitating conditions like OA [16].

Given the current challenges of identifying disease-modifying therapeutic strategies for patients with OA [17], novel alternatives are currently under clinical investigation including cell-based therapy approaches that have yielded encouraging initial results. For instance, early-stage clinical trials using either heterogeneous adipose-derived stromal vascular fraction cells [18] or expanded mesenchymal stem/stromal cells (MSC) derived from either umbilical cord [19] or bone marrow [20] have demonstrated clinical superiority when compared with current alternatives such as hyaluronic acid intra-articular placement. Mechanistically, when exposed to an inflammatory environment, MSC exert “medicinal signaling” activities [21] due to their sensory capacity and secretion of immunomodulatory mediators such as the tryptophan depleting enzyme indoleamine 2,3-dioxygenase (IDO), interleukin-10 (IL-10), and prostaglandin E_2_ (PGE2) [22, 23], thereby resulting in strong anti-inflammatory effects. However, multiple clinical trials still show only moderate or even inconsistent results. To help address this limitation, preclinical studies have shown that MSC can be “functionalized” in vitro [24] to enhance their therapeutic capacities, while simultaneously reducing the intrinsic phenotypic and functional variabilities within cell preparations.

MSC can be extracted from multiple sources including the knee’s synovium and IFP [25, 26]. IFP-MSC constitute a promising treatment vehicle given their local presence within the joint, ease of harvest during knee arthroscopy, and high proliferation rate in vitro [25, 27]. To that end, we have shown that IFP-MSC possess an intrinsic immunomodulatory secretory profile involving in vitro and in vivo efficient degradation of the nociception and inflammation regulator substance P (SP) through a CD10 (neprilysin/NEP)-dependent pathway. Collectively, this cascade of events leads to reversal of synovial and IFP inflammation and fibrosis. Importantly, these specific attributes and functions can be induced or further enhanced in vitro prior to the administration of the cells. On this basis, we have reported that IFP-MSC exposed to inflammatory cues including TNF-α, IFN-γ, and CTGF (i.e., cell priming/licensing) and/or expanded under regulatory-compliant conditions (e.g., pooled human platelet lysate—hPL) can effectively reinforce the critical CD10^+^ phenotype, which translates into enhanced functional properties both in vitro and in vivo [28–30].

On the other hand, we have also recently reported that a similar inflammatory cell priming protocol applied to bone marrow-derived MSC (BM-MSC) enriches the preparation in CD146^+^ cells, unveiling a subset with innately higher immunomodulatory and secretory capacities compared to their CD146^−^ counterparts [31]. To the best of our knowledge, a comparable CD146-dependent phenotypic and functional discrimination in IFP-MSC has not been reported to date. Consequently, coupling CD146 with CD10-dependent phenotype-based MSC purification of heterogeneous preparations could result in cell products with combined enhanced biological functions, as previously shown for other defining markers such as CD271 [32, 33].

MSC possess a remarkable ability to coalesce and assemble in tri-dimensional (3D) structures (i.e., MSC spheroids) that closely recapitulate the in vivo MSC niche by providing spatial cell organization with increased cell-cell interactions. In that context, 3D spheroids provide MSC a stable immunophenotypic profile, with reinforced survival, homing, stemness, differentiation potential, angiogenic, and anti-inflammatory properties [34]. MSC-based spheroids have been applied in various preclinical models including wound healing, bone and osteochondral defects, and cardiovascular diseases while at the same time demonstrating safety and efficacy (reviewed in [24]).

The current study explores the effects on the phenotypic, transcriptional, secretory, and functional IFP-MSC profiles when manufactured as 3D spheroids and following phenotype-based MSC purification. These data further expand our understanding of IFP-MSC responses to inflammatory/fibrotic environments. The resulting evidence could be harnessed as a foundation for the design of novel and/or to modify existing clinical protocols using IFP-MSC for joint inflammatory disease treatment, with potentially more reproducible clinical outcomes.

## Methods

### Cell and animal protocols

IFP-MSC were isolated from IFP tissue obtained from de-identified, non-arthritic patients (seven males and six females, with an age range between 17 and 60 years old) undergoing elective knee arthroscopy at the Lennar Foundation Medical Center–University of Miami and after provided written informed consent. All procedures were carried out in accordance with relevant guidelines and regulations and following a protocol determined by the University of Miami IRB not as human research (based on the nature of the samples as discarded tissue). IFP tissue (< 20 ml) was mechanically dissected and washed repeatedly with Dulbecco’s Phosphate Buffered Saline (PBS; Sigma), followed by enzymatic digestion using 235 U/ml Collagenase I (Worthington Industries, Columbus, OH) diluted in PBS and 1% bovine serum albumin (Sigma) for 2 h at 37 °C with agitation. Cell digests were inactivated with complete media [DMEM low glucose GlutaMAX (ThermoFisher Scientific, Waltham, MA) + 10% fetal bovine serum (FBS; VWR, Radnor, PA)], washed and seeded at a density of 1 × 10^6^ cells/175 cm^2^ flask in complete media. Medium was changed 2 days after cell seeding. Plastic adherent IFP-MSC were cultured at 37 °C 5% (v/v) CO_2_ until 80% confluent (denoted as P0), then passaged at a 1:5 ratio until P3 detaching them with TrypLE™ Select Enzyme 1× (Gibco, ThermoFisher Scientific) and assessing cell viability with 0.4% (w/v) Trypan Blue (Invitrogen, Carlsbad, CA).

The animal protocol was approved by the Institutional Animal Care and Use Committee (IACUC) of the University of Miami, USA (approval no. 16-008-ad03), and conducted in accordance to the ARRIVE guidelines. Sixteen (#16) 10-week-old Sprague Dawley rats (8 males and 8 females; mean weight 250 g and 200 g, respectively) were used. The animals were housed to acclimate for 1 week before the experiment initiation. One rat was housed per cage in a sanitary, ventilated room with controlled temperature, humidity, and under a 12/12-h light/dark cycle with food and water provided ad libitum.

### Culture of IFP-MSC spheroids and synoviocytes

IFP-MSC spheroids were created using gas-permeable culture plates (Miltenyi Biotech, Inc., Auburn, CA). Briefly, 2 × 10^5^ cells resuspended in DMEM/10%FBS + methylcellulose solution (4/1 ratio) were seeded per well of a 6-well gas-permeable culture plate and cultured for 2 days at 37 °C and 5% CO_2_. MSC spheroids were evaluated for phenotypic, secretory, transcriptional, and functional profiles.

Synoviocytes (Sciencell, Carlsbad, USA) were cultured using synoviocyte medium (Sciencell) at 37 °C 5% (v/v) CO_2_ until 80% confluent (denoted as P0), then passaged at a 1:4 ratio until P2 detaching them with TrypLE™ Select Enzyme 1× (Gibco, ThermoFisher Scientific) and assessing cell viability with 0.4% (w/v) Trypan Blue (Invitrogen, Carlsbad, CA).

### MSC and synoviocyte priming

P3 Crude IFP-MSC (*n* = 5) were seeded in 2D or 3D settings in 6-well plates at a density of 2 × 10^5^ cells/well in complete medium. Next day (24 h), cultures were primed with TI inflammatory cocktail (15 ng/ml TNFα, 10 ng/ml IFNγ) for 48 h or TIC inflammatory/fibrotic cocktail (15 ng/ml TNFα, 10 ng/ml IFNγ, 10 ng/ml CTGF) for 72 h. Non-induced and both TI- and TIC-primed cohorts were evaluated for secretory and transcriptional profiles. Non-induced and TIC-primed cohorts were evaluated for functional profiles.

### CD146 surface marker-based magnetic immunoselection

Immunomagnetic cell sorting was performed in P1 Crude IFP-MSC (*n* = 5). Briefly, 2 × 10^6^ cells Crude IFP-MSC were resuspended in 1× PBS with 0.5% bovine serum albumin (BSA) and 2 mM EDTA and incubated with biotinylated anti-human CD146 (Miltenyi Biotech) at 4 °C for 20 min with agitation. Invitrogen™ CELLection Dynabeads™ Biotin Binder Kit (ThermoFisher Scientific) was used for magnetic immunoselection resulting in the CD146POS and CD146NEG subpopulations according to the manufacturer’s instructions. The generated P2 CD146POS and P2 CD146NEG subpopulations were directly plated and expanded with DMEM/10%FBS until 70–80% confluency. All IFP-MSC subpopulations were stored in liquid nitrogen until further experimentation.

### Immunophenotype

Flow cytometric analysis was performed on P3 naïve IFP (*n* = 3) MSC. 2.0 × 10^5^ cells were labeled with fluorochrome-conjugated monoclonal antibodies specific for CD10, CD44, CD56, CD90 (Biolegend, San Diego, CA), CD146 (Miltenyi Biotech), NG2 (BD Biosciences, San Jose, CA), CXCR4 (Invitrogen), and the corresponding isotype controls. Immunophenotyping marker selection was based on our previous data which associate their expression levels with distinct immunomodulatory signatures and functionalities. All samples included a Ghost Red Viability Dye (Tonbo Biosciences, San Diego, CA). Data were acquired using a Cytoflex S (Beckman Coulter, Brea, CA) and analyzed using Kaluza analysis software (Beckman Coulter).

Immunofluorescence was performed on P3 IFP-MSC spheroids (*n* = 3) in suspension cultures. Briefly, IFP-MSC spheroids were fixed in 3.7% paraformaldehyde for 1 h at RT, permeabilized with 0.2% Triton-X/gelatin solution for 1 h, and subsequently with 0.5% Triton-X/gelatin solution for 15 min. Fixed/permeabilized IFP-MSC spheroids were incubated with unconjugated primary antibodies CD10, CD44, CD90 (Abcam, Cambridge, MA), CD146 (Miltenyi Biotech), CXCR4 (Abcam), and NG2 (Invitrogen) overnight at 4 °C. Next day, the spheroids were washed 5× with 0.2% Triton-X and incubated with secondary antibodies for 1 h. After rinsing 5× with 0.2% Triton-X and incubation with DAPI (Invitrogen) for 10 min, images were captured on a Leica TCS SP5 confocal microscope using × 20 objective and evaluated with ImageJ software. All quantifications were performed in at least 5 regions of interest (ROIs) per IFP-MSC spheroid and in total 5 spheroids per IFP-MSC spheroid subpopulation. Quantitations were performed using the formula: corrected total cell fluorescence (CTGF) = integrated density − (area of selected cell × mean fluorescence of background readings).

### Quantitative real-time PCR (qPCR)

RNA extraction was performed using the RNeasy Mini Kit (Qiagen, Frederick, MD) according to the manufacturer’s instructions. Total RNA (1 μg) was used for reverse transcription with SuperScript™ VILO™ cDNA synthesis kit (Invitrogen), and 10 ng of the resulting cDNA was analyzed by qPCR using QuantiFast SYBR Green qPCR kit (Qiagen) and a StepOne Real-time thermocycler (Applied Biosystems, Foster City, CA). For each target, human transcript primers were selected using PrimerQuest (Integrated DNA Technologies, San Jose, CA) (Supplementary Table S1). All samples were analyzed in triplicate. Mean values were normalized to GAPDH, and expression levels were calculated using the 2^−ΔΔ^Ct method and represented as the relative fold change of the primed cohort to the naïve (= 1).

A pre-designed 90 gene Taqman-based mesenchymal stem cell qPCR array (Stem Cell Technologies, Supplementary Table S2) was performed (*n* = 3) using 1000 ng cDNA per IFP-MSC sample and processed using StepOne Real-time thermocycler (Applied Biosystems). Data analysis was performed using Stem Cell Technologies qPCR online analysis tool (Stem Cell Technologies). Sample and control Ct values were expressed as 2^−ΔΔ^Ct (with 38 cycles cutoff point). The expression levels were represented in bar plots ranked by transcript expression levels on a log-transformed scale of sample compared to control cohorts. Bar plots were color-coded by the functional class of genes (namely Stemness, MSC, MSC-related/Angiogenic, Chondrogenic/Osteogenic, Chondrogenic, Osteogenic, Adipogenic). A *t* test (unpaired, two-tailed test with equal variance) is used in all statistical analysis, and *p* values were corrected for multiple comparisons by the Benjamini-Hochberg procedure. Two groups were compared and presented in bar plots: sample (spheroids) versus control (2D cultures), and sample (CD146POS spheroids) versus control (Crude spheroids).

### Trilineage differentiation

Osteogenic, chondrogenic, and adipogenic differentiation potential was evaluated in P3 IFP-MSC spheroids (*n* = 3) similar to previously published protocols [35]. Briefly, MSC spheroids were created using gas-permeable culture plates (Miltenyi Biotech) and methylcellulose and cultured for 2 days at 37 °C and 5% CO_2_ as described above. On day 2, IFP-MSC spheroids were subsequently induced towards osteogenesis, adipogenesis, and chondrogenesis by changing the medium to induction media in the separate gas-permeable culture plate wells. Chondrogenic differentiation was induced for 21 days with serum-free MesenCult-ACF differentiation medium (STEMCELL Technologies Inc., Vancouver, Canada). Harvested spheroids were cryosectioned and 4-μm frozen sections stained with 1% toluidine blue (Sigma) for semi-quantitative assessment of chondrogenic differentiation. Osteogenic differentiation was induced for 21 days with StemPro Osteogenesis differentiation kit (ThermoFisher Scientific). Harvested spheroids were cryosectioned and 4-μm frozen sections stained with 1% Alizarin Red S (Sigma) for semi-quantitative assessment of mineralization. Adipogenic differentiation was induced for 21 days with StemPro Adipogenesis kit (ThermoFisher Scientific). Harvested spheroids were cryosectioned and 4-μm frozen sections stained with 0.5% Oil Red (Sigma) for semi-quantitative assessment of lipid accumulation within the cell cytoplasm. Differentiation status of 3D IFP-MSC spheroids was evaluated by the expression levels of specific differentiation-related transcripts using qPCR (Supplementary Table S1).

### Secretome analysis

Arrays for growth factors (GFs) and inflammatory mediators (RayBio® C-Series, RayBiotech, Peachtree Corners, GA) were used to determine secreted levels obtained from P3 Crude 2D IFP-MSC, and P3 Crude and CD146-selected IFP-MSC spheroids pre- and post-priming. For each population, 1 ml of conditioned media obtained from 2 donors was prepared and used for each assay following the manufacturer’s instructions. Data shown represent 40 s exposure in FluorChem E chemiluminescence imaging system (ProteinSimple, San Jose, CA). Results were generated by quantifying the mean spot pixel density of each array using protein array analyzer plugin using ImageJ software (Fiji/ImageJ, NIH website). The signal intensities were normalized with the background whereas separate signal intensity results represent the average pixel density of two spots per protein. The signal intensity for each protein spot is proportional to the relative concentration of the antigen in the sample. The protein levels contained in the medium used were subtracted from condition media protein levels in order to obtain the actual samples’ secreted levels.

### Pathway analysis

Putative interactomes were generated by Search Tool for Retrieval of Interacting Genes/Proteins (STRING 11.0; available from: http://string-db.org) database using interaction data from experiments, databases, neighborhood in genome, gene fusions, co-occurrence across genomes, co-expression, and text-mining. An interaction confidence score of 0.4 was imposed to ensure high interaction probability. *K*-means clustering algorithm was used to organize proteins into 3 separate clusters per condition tested, discriminated by colors. Venn diagrams were used to demonstrate all possible relations between Crude and CD146-selected IFP-MSC subpopulations post-TI and post-TIC priming for the significantly (*p* < 0.05) altered proteins. Functional enrichments related to biological process, Kyoto Encyclopedia of Genes and Genomes (KEGG) pathways, and reactome pathways were presented in radar graphs for all conditions tested. Seven biological processes were evaluated namely positive regulation of cell population proliferation (GO:0008284), positive regulation of response to stimulus (GO:0048584), positive regulation of cell migration (GO:0030335), regulation of signal transduction (GO:0009966), regulation of signaling receptor activity (GO:0010469), positive regulation of protein phosphorylation (GO:0001934), and angiogenesis (GO:0001525). Additionally, eight KEGG reactome pathways were evaluated including cytokine-cytokine receptor interaction (hsa:04060), MAPK signaling (hsa:04010), PI3K-Akt signaling (hsa:04151), Ras signaling (hsa:04014), Jak-STAT signaling (has:04630), Rap1 signaling (has:04015), signaling by interleukins (HSA: 449147), and interleukin-10 signaling (HSA: 6783783).

### Indoleamine 2,3-dioxygenase (IDO) and prostaglandin E_2_ in vitro assay

IDO SimpleStep ELISA kit (Abcam, MA, USA) and Parameter Prostaglandin E_2_ (PGE_2_) competitive immunoassay (R&D Systems, MN, USA) were used to quantify the secreted levels (pg/ml) in P3 Crude and CD146-selected IFP-MSC spheroids with and without TIC priming (10^5^ IFP-MSC/well, 12-well; *n* = 3 per cohort), following the manufacturer’s instructions. IDO and PGE_2_ secretions were quantified in centrifuged (1500 rpm; 5 min) conditioned media (run in duplicates) obtained from IFP-MSC cultures in all conditions. Levels were determined by measuring the fluorescence (450 nm) of individual wells in end-point mode (SpectraMax M5 spectrophotometer, Molecular Devices, San Jose, CA, USA). Secretion was normalized to total protein secreted per cohort.

### Immunopotency assay (IPA)

P3 Crude and CD146-selected IFP-MSC spheroids (*n* = 3) were designated into non-induced or TIC-primed cohorts. After 72 h in complete culture (non-induced) or TIC priming (primed) media, IFP-MSC cultures were washed once with PBS and media were changed to IPA medium containing RPMI (Gibco) with 15% Human AB Serum (Corning, Corning, NY, USA), 1% l-glutamine (Gibco), 1% Non-Essential Amino Acids (Gibco), 1% Sodium Pyruvate, 1% HEPES (Gibco), and 1% 100X Vitamins (Gibco). Human peripheral blood mononuclear cells (PBMC, 3 male and 3 female donors) (Continental Services Group Blood Bank, Miami, USA) were stained with CellTrace™ CFSE Cell Proliferation Kit (Invitrogen) according to the manufacturer’s instructions and resuspended in IPA media. PBMC/IFP-MSC non-contact co-cultures were performed by seeding CFSE-stained PBMC in the lower chamber of a 12-well plate and non-induced or TIC-primed IFP-MSC spheroids in transwells (0.4 μm pore size, Corning) in IPA medium at a 2:1 ratio. Cell stimulation cocktail 500× (Invitrogen) containing phorbol 12-myristate 13-acetate and ionomycin was then added to the wells designated for PBMC stimulation. After 96 h, CFSE-stained cells were collected, stained with Ghost Red 780 Viability Dye (Tonbo Biosciences), and acquisition of 10,000 was performed using a CytoFLEX S cytometer with CytExpert software (Beckman Coulter). PBMC were gated by scatter, viability, and CFSE positivity, and PBMC proliferation was represented as CFSE^dim^/total CFSEx100.

### IFP-MSC/synoviocyte co-cultures

P3 synoviocytes were seeded in 6-well plates at a density of 2 × 10^5^ cells/well in synoviocyte medium. Next day (24 h), synoviocyte cultures were designated into non-induced or TIC-primed cohorts and cultured for 3 days with the appropriate medium. On day 3, synoviocyte/IFP-MSC co-cultures were performed by seeding non-induced IFP-MSC spheroids on top of synoviocyte monolayers at a density of 5 × 10^5^ cells/well. Co-cultures were fed with synoviocyte medium or synoviocyte medium + TIC inflammatory/fibrotic cocktail (15 ng/ml TNFα, 10 ng/ml IFNγ, 10 ng/ml CTGF) for 72 h according to the initial non-induced or TIC-primed cohorts designation. The synoviocyte secretory and transcriptional profiles were evaluated in both non-induced and primed cohorts. Microscope images of IFP-MSC/synoviocyte co-cultures were acquired using × 10 objective Leica DMi8 microscope with Leica X software (Leica).

### Mono-iodoacetate model of acute synovial/IFP inflammation

Acute synovial/IFP inflammation was generated by intra-articular injection of 1 mg of mono-iodoacetate (MIA) in 50 μl of saline in the right knee. Briefly, under isoflurane inhalation anesthesia, rat knees were flexed 90° and MIA was injected into the medial side of the joint with a 27G needle using the patellar ligament and articular line as anatomical references. This short exposure to MIA has been shown to induce inflammatory changes at the synovium and adjacent IFP [36]. Three (3) days later, a single intra-articular injection of 500,000 IFP-MSC spheroids in 50 μl of Euro-Collins solution (MediaTech) was performed (similar injection technique), having as control: (1) rats receiving MIA but not IFP-MSC (Only MIA group) and (2) healthy rats receiving only IFP-MSC spheroids (Only IFP-MSC group). Animals were sacrificed at two different timepoints: 4 and 25 days after IFP-MSC injection (day 7 and day 28 total, respectively).

### Tissue preparation and histological analysis

Rat knee joints were harvested by cutting the femur and tibia/fibula 1 cm above and below the joint line, muscles were removed, and joints were fixed with 10% neutral buffered formalin (Sigma-Aldrich) for 14 days at room temperature. Knee joints were decalcified, cut at sagittal plane in half, and embedded in paraffin, and serial 4-μm sections were obtained. Hematoxylin and eosin (H&E) staining was performed to evaluate the structure and morphology of knee joints. Masson’s trichrome staining for collagen-rich fibrotic areas was used to evaluate the extent of fibrosis in fat pad tissue. Toluidine blue staining for cartilage proteoglycan subunits was used to evaluate articular cartilage quality changes. Microscope images of cytochemically stained tissues were acquired using × 10 and × 20 objectives Leica DMi8 microscope with Leica X software (Leica). Based on histochemical stainings, tissue synovitis/fibrosis was evaluated in 3 rat knees per condition and 4 microscopy fields per knee with ImageJ software.

For anti-substance P immunofluorescence staining, sections were incubated with 1× citrate buffer solution at 60 °C overnight for antigen retrieval, permeabilized with 1× PBS + 0.2% Triton X-100 for 20 min at room temperature, and incubated with blocking buffer (1× PBS + 0.1% Triton X-100 with 10% rabbit serum) for 1 h at room temperature. In between different treatments, sections were washed with 1× PBS. Rabbit anti-rat substance P polyclonal antibody (Millipore) was prepared in blocking buffer (1:100), and sections were incubated at 4 °C overnight. Sections were washed with 1× PBS + 0.01% Triton X-100 and incubated for 1 h with secondary antibody containing Alexa Fluor594 conjugated goat anti-rabbit IgG antibody (ThermoFisher Scientific) at room temperature. Controls were incubated with secondary antibody only. All sections were rinsed with 1× PBS, mounted in prolong gold antifade reagent with DAPI (Invitrogen), and microscope images were acquired using × 10 objective Leica DMi8 microscope with Leica X software (Leica). Substance P tissue distribution was evaluated in 3 rat knees per condition and 4 microscopy fields per knee with ImageJ software.

### Statistical analysis

Statistical analysis was performed using paired and unpaired Student’s *t* test for normally distributed data and Wilcoxon (for paired data) or Mann-Whitney (for unpaired data) test in presence of a non-normal distribution; one-way ANOVA was used for multiple comparisons. All tests were performed with GraphPad Prism v7.03 (GraphPad Software, San Diego, CA). Level of significance was set at *p* < 0.05. Data used for the statistical analyses is indicated in the figure legends, overall corresponding to three independent experiments from different MSC donors (*n* = 3), unless specified.

## Results

### Phenotypic characterization of 2D IFP-MSC and IFP-MSC spheroids

**Fig. 1:**
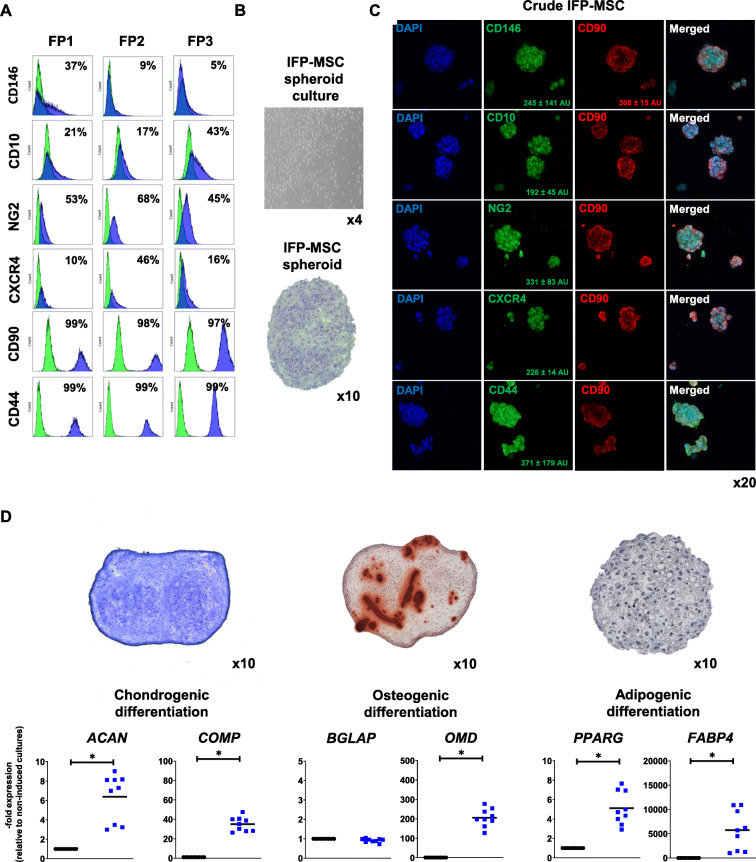
IFP-MSC spheroid immunophenotypic profile and tripotentiality. **a** In 2D settings, Crude IFP-MSC show stable MSC-related phenotypic profile with high expression levels for CD44 and CD90 markers and increased donor-to-donor variability in CD10, CD146, and CXCR4 expression levels. **b**, **c** Upon seeding in gas-permeable culture plates, Crude IFP-MSC can generate multiple sized spheroids (100–300 μm) with enhanced expression of CD90, CXCR4, CD10, and NG2. CD146 and CD44 show variable expression levels between spheroids. **d** IFP-MSC spheroids can efficiently differentiate towards chondrogenic, osteogenic, and adipogenic lineages after specific induction in gas-permeable plates. Quantitative molecular profiling showed that differentiation-related markers in induced IFP-MSC spheroids were increased compared to non-induced cultures, indicating their mature status. All experiments (*n* = 3) were performed independently, and data are presented as mean ± SD (*p < 0.05)

IFP-MSC expanded in 2D cultures showed the characteristic fibroblast-like morphology with high (> 95%) viability in between different passages. A complete immunophenotyping of IFP-MSC expanded in DMEM/10%FBS and other regulatory-complaint formulations have been previously reported in [28–30]. Herein, we confirm the stable MSC-related phenotypic profile of IFP-MSC expanded in 2D cultures (Fig. 1a).

Crude IFP-MSC seeding with methylcellulose in gas-permeable culture plates for 2 days results in the generation of multiple sized (100–300 μm) non-adherent IFP-MSC spheroids which show compact spheroid intra-structural organization with increased cell-cell interactions and no necrotic core, implying the physiological nutrient and gas supply reaching the center of every individual spheroid (Fig. 1b). Immunophenotypic profiling of the generated spheroids revealed enhanced and similar between different spheroids expression of CD90 (308 *±* 15 AU), CXCR4 (226 *±* 14 AU), CD10 (192 *±* 45 AU), and NG2 (331 *±* 83 AU). Interestingly, CD146 show high expression levels but variable between different spheroids (245 *±* 141 AU) whereas CD44 showed also high variability (371 *±* 179 AU) (Fig. 1c).

In order to evaluate the differentiation capacity of generated IFP-MSC spheroids, we have performed tripotentiality assessment in vitro showing that upon specific induction in gas-permeable plates, IFP-MSC spheroids can efficiently differentiate towards chondrogenic, osteogenic, and adipogenic lineages (Fig. 1d). Quantitatively at the molecular level, Crude IFP-MSC spheroids show significantly (*p* < 0.05) higher expression levels for chondrogenic genes *ACAN* and *COMP*, the osteogenic gene *OMD*, and adipogenic genes *FABP4* and *PPARγ* compared to non-induced controls, indicating their increased maturity during the different differentiation schemes.

### Molecular and secretory profiles comparison of 2D IFP-MSC and IFP-MSC spheroids

**Fig. 2:**
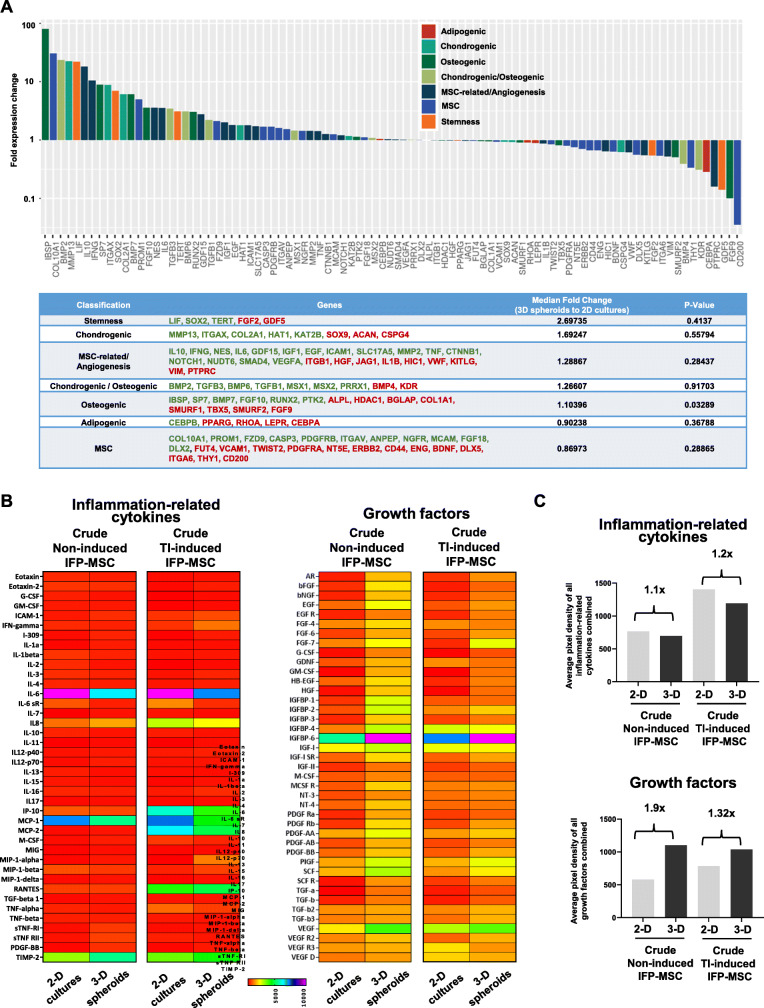
Molecular and secretory profiling of 2D IFP-MSC and IFP-MSC spheroids. **a** Molecular profiling of non-induced IFP-MSC spheroids versus non-induced 2D IFP-MSC cultures revealed that 49 out of 90 genes tested were higher expressed in IFP-MSC spheroids with 24 genes being more than twofold higher. Genes tested were grouped in phenotype/function-related cohorts. **b**, **c** The secretory profile of inflammation-related cytokines revealed that non-induced IFP-MSC spheroids show significantly (*p* < 0.05) higher secretion of 13 and lower secretion of 5 (IL-6, IL-6sR, IL-16, MIP-1-β, TNF-α) out of 40 secreted proteins tested compared to naïve 2D IFP-MSC cultures. In addition, non-induced IFP-MSC spheroids show significantly (*p* < 0.05) higher secretion of 36 out of 41 secreted protein tested. Heat maps’ colors are assigned according to a molecule concentration relative scale, from 0 to 10,000. All experiments were performed independently (*n* = 2)

Molecular profiling of IFP-MSC spheroids versus 2D IFP-MSC cultures revealed that 49 out of 90 genes tested were higher expressed in IFP-MSC spheroids with 24 genes being more than twofold higher (*IBSP*, *COL10A1*, *BMP2*, *MMP13*, *LIF*, *IL10*, *IFNG*, *SP7*, *ITGAX*, *SOX2*, *COL2A1*, *BMP7*, *PROM1*, *FGF10*, *NES*, *IL6*, *TGFB3*, *TERT*, *BMP6*, *RUNX2*, *GDF15*, *TGFB1*, *FZD9*, *IGF1*). Interestingly, genes tested were grouped in phenotype/function-related cohorts with stemness cohort showing overall the most prominent fold expression change between IFP-MSC spheroids and 2D IFP-MSC cultures (Fig. 2a).

The secretory profile of inflammation-related cytokines revealed that IFP-MSC spheroids show significantly (*p* < 0.05) higher secretion of 13 and lower secretion of 5 (IL-6, IL-6sR, IL-16, MIP-1-β, TNF-α) out of 40 secreted proteins tested compared to 2D IFP-MSC cultures (Fig. 2b). Among the highly secreted proteins in IFP-MSC spheroids were IL-1α, IL-7, IL-8, IL-11, IL-12-p70, M-CSF, MIG, MIP-1-α, MIP-1-δ, sTNF-RI, sTNF-RII, PDGF-BB, and TIMP-2. Interestingly, spheroid culturing of IFP-MSC results in lower secretion of IL-6 and higher secretion of IL-8 protein, which associates with significantly lower IL-6/IL-8 ratio suggesting a less pro-inflammatory status compared to 2D IFP-MSC cultures. Upon TI induction, IFP-MSC spheroids showed increased secretion of 11 (Eotaxin-2, G-CSF, GM-CSF, IFN-γ, I-309, IL-2, IL-11, MIP-1-α, RANTES, PDGF-BB, and TIMP-2) and decreased secretion of 6 (IL-6, IL-6sR, IP-10, MCP-1, MCP-2, TNF-α) proteins compared to 2D IFP-MSC cultures. Importantly, the overall protein secretion of inflammation-related cytokines in IFP-MSC spheroids is reduced compared to 2D IFP-MSC cultures in both non-induced and TI-induced conditions (1.1-fold and 1.2-fold, respectively; Fig. 2c).

In contrast, IFP-MSC spheroids strongly secrete reparative growth factors compared to 2D IFP-MSC cultures even without TI induction (Fig. 2b). Specifically, IFP-MSC spheroids show significantly (*p* < 0.05) higher secretion of 36 out of 41 secreted protein tested. Upon TI induction, the amount of significantly higher secreted proteins in IFP-MSC spheroids is reduced as 2D IFP-MSC cultures increase their overall secretory profile. Therefore, the overall IFP-MSC spheroid growth factor secretory profile is enhanced compared to 2D IFP-MSC cultures in both non-induced and TI-induced conditions (1.9-fold and 1.32-fold, respectively; Fig. 2c).

### Crude and CD146-selected IFP-MSC spheroids show distinct phenotypic and molecular profiles

**Fig. 3:**
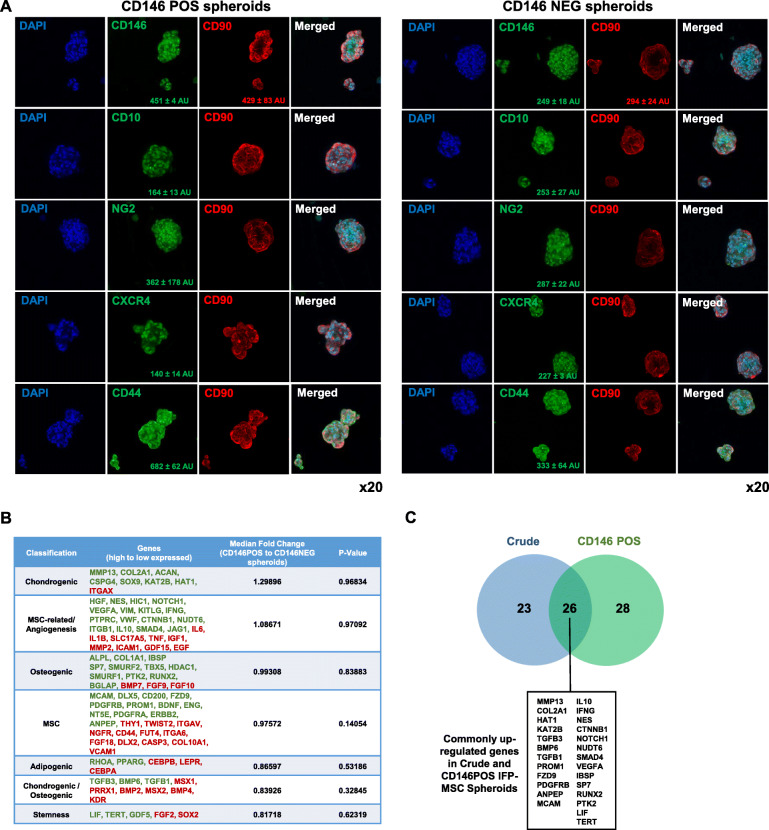
Immunophenotype and molecular profile of CD146-selected IFP-MSC spheroids. **a** Upon seeding in gas-permeable culture plates, CD146POS IFP-MSC spheroids show as expected higher expression levels of CD146 (451 *±* 4 AU vs 241 *±* 18 AU) and higher expression levels of NG2 (362 *±* 178 AU vs 287 *±* 22 AU), whereas CD146NEG IFP-MSC spheroids show higher expression levels of CD10 (253 *±* 27 AU vs 164 *±* 13 AU) and CXCR4 (227 *±* 3 AU vs 140 *±* 14 AU). **b** Molecular profiling of CD146POS versus CD146NEG IFP-MSC spheroids revealed that 55 out of 90 genes tested were higher expressed in CD146POS IFP-MSC spheroids with 12 genes being more than twofold higher (*MCAM*, *HGF*, *ALPL*, *DLX5*, *NES*, *MMP13*, *CD200*, *COL1A1*, *FZD9*, *PDGFRB*, *HIC1*). Genes tested were grouped in phenotype/function-related cohorts. **c** A total of 26 genes are shared between Crude and CD146POS spheroids, indicating similarities in their molecular signatures. However, upon TI or TIC induction, most of the immunoregulatory-related genes tested show upregulated expression levels in CD146POS spheroids. All experiments were performed independently (*n* = 3)

CD146-based magnetic immunoselection of Crude IFP-MSC resulted in the generation of two separate subpopulations (CD146POS and CD146NEG) showing significant different CD146 protein levels in 2D cultures and distinct immunophenotypic profiles in 3D settings (Supplementary Figure 1A and Fig. 3a). Specifically, CD146POS IFP-MSC spheroids show as expected higher expression levels of CD146 (451 *±* 4 AU vs 249 *±* 18 AU) and higher expression levels of NG2 (362 *±* 178 AU vs 287 *±* 22 AU) compared to CD146NEG IFP-MSC spheroids, indicating their enhanced pericytic phenotype (Fig. 3a, left panel). Interestingly, *CD146* gene expression is not absent in CD146NEG IFP-MSC spheroids suggesting that 3D spheroid culturing in vitro can positively affect *CD146* gene expression levels (Supplementary Figure 1A). Importantly, the CD146 protein presence is coupled with gene expression, which shows not only its upregulation in 3D settings but also significant expression also in the CD146NEG cohort (Fig. 3b). In parallel, CD146NEG IFP-MSC spheroids show higher expression levels of CD10 (253 *±* 27 AU vs 164 *±* 13 AU) and CXCR4 (227 *±* 3 AU vs 140 *±* 14 AU) compared to CD146POS IFP-MSC spheroids (Fig. 3a, right panel). Putatively, low expression levels of surface bound CD10 in CD146POS IFP-MSC spheroids are correlated with high CD10 secretion capacity as we previously reported [28]. Interestingly, in CD146POS, CD44 (682 *±* 62 AU vs 333 *±* 64 AU) and CD90 (429 *±* 83 AU vs 294 *±* 24 AU) had higher expression levels compared to CD146NEG IFP-MSC spheroids, possibly associated with their pericytic phenotype and increased stemness in vitro.

Molecular profiling of CD146POS versus CD146NEG IFP-MSC spheroids revealed that 55 out of 90 genes tested were higher expressed in CD146POS IFP-MSC spheroids with 12 genes being more than twofold higher (*MCAM*, *HGF*, *ALPL*, *DLX5*, *NES*, *MMP13*, *CD200*, *COL1A1*, *FZD9*, *PDGFRB*, *HIC1*). Interestingly, genes tested were grouped in phenotype/function-related cohorts with chondrogenesis and MSC-related/angiogenesis cohorts showing overall the most prominent fold expression change between CD146POS and CD146NEG cohorts (Fig. 3c). By comparing the significantly upregulated genes in Crude spheroids versus 2D cultures (Fig. 2a) and the significantly upregulated genes in CD146POS versus CD146NEG cohorts, 26 genes are shared between Crude and CD146POS spheroids, indicating similarities in their molecular signatures (Fig. 3d). However, upon TI or TIC induction, most of the immunomodulatory-related genes tested show upregulated expression levels in CD146POS spheroids (Supplementary Figure 1B).

### Crude and CD146-selected IFP-MSC spheroids exhibit a robust secretion of reparative growth factors with and without exposure to priming inflammatory/fibrotic cues

**Fig. 4:**
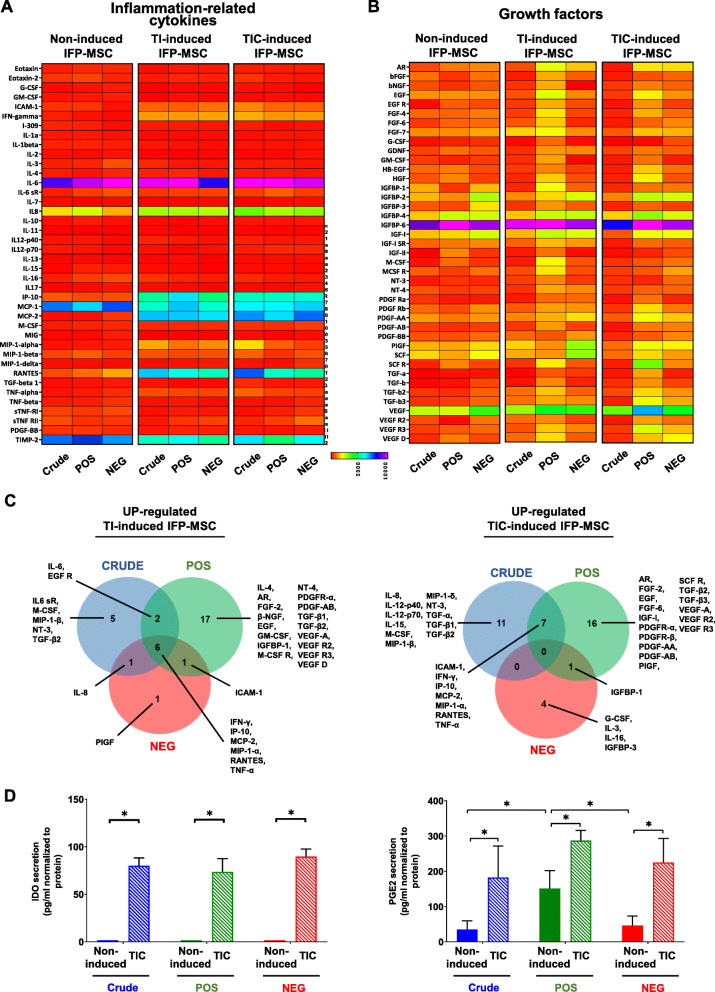
Secretory profiling of Crude and CD146-selected IFP-MSC spheroids with and without exposure to inflammatory/fibrotic cues. **a**, **b** Crude and CD146-selected IFP-MSC spheroids show similar inflammation-related secretory profiles both in non-induced and TI- or TIC-induced conditions. However, their secretory profiles of reparative growth factors were distinct and characterized by the secretion of different numbers and types of proteins. Heat maps’ colors are assigned according to a molecule concentration relative scale, from 0 to 10,000 (*n* = 2). **c** In both TI- and TIC-induced conditions, CD146POS IFP-MSC spheroids show higher number of secreted proteins. Among the proteins with upregulated secretion levels, 6 and none proteins are commonly shared between the three different cohorts (Crude, CD146POS, CD146NEG) after TI and TIC induction, respectively. Venn diagram showing shared proteins among the three different cohorts (Crude, CD146POS, CD146NEG) after TI and TIC induction. All experiments were performed independently (*n* = 2). **d** In non-induced cohorts, IDO secretion is almost absent whereas PGE2 secretion is evident in all three cohorts. TIC induction results in increased IDO and PGE2 secretion levels (*p < 0.05, *n* = 3)

Crude and CD146-selected IFP-MSC spheroids show similar inflammation-related secretory profiles both in non-induced and TI- or TIC-induced conditions. However, their secretory profiles of reparative growth factors were distinct and characterized by the secretion of different numbers and types of proteins (Fig. 4a, b). Specifically, in non-induced conditions, when comparing the CD146POS with Crude IFP-MSC spheroids, 6 proteins show significantly higher (FGF-6, EGF R, IGFBP-4, IGFBP-6, IGF-I sR, and IL-6 sR; *p* < 0.05) and 5 proteins significantly lower (βFGF, IGFBP-1, SCF, PDGF-AB, and IL-4; *p* < 0.05) secretion levels in CD146POS spheroids. Upon TI induction, CD146POS IFP-MSC spheroids show higher number of proteins with upregulated (26) and lower number of proteins with downregulated (1) secretion levels compared to other spheroid cohorts (Supplementary Figure 2A). Interestingly, among the proteins with upregulated secretion levels after TI induction, 6 proteins were commonly shared between the three different cohorts (Fig. 4c). A similar pattern was observed in all three different cohorts after TIC induction with CD146POS IFP-MSC spheroids, having upregulated secretion of 24 proteins and downregulated secretion of only 1 protein (Supplementary Figure 2B). However, among the proteins with upregulated secretion levels after TIC induction, none are shared among the three different cohorts (Crude, CD146POS, CD146NEG), putatively indicating further secretory profile distinction between cohorts (Fig. 4c).

In TI-induced IFP-MSC spheroids, all subpopulation cohorts revealed overall similar biological processes involvement (Supplementary Figure 3A). In TIC-induced IFP-MSC spheroids, Crude cohort showed higher involvement in 4 out of 7 biological processes tested (Supplementary Figure 3B). Overall, in both TI- and TIC-induced IFP-MSC spheroids, CD146POS cohort showed significantly stronger protein involvement in 4 out of 8 KEGG reactome pathways (MAPK, PI3K-Akt, Ras, and Rap1 signaling pathways) compared to other cohorts.

In non-induced cohorts, IDO secretion was almost absent whereas TIC-induced IFP-MSC spheroids showed significantly (*p* < 0.05) higher IDO secretion (80-fold for Crude, 73-fold for CD146POS, and 89-fold for CD146NEG; Fig. 4d). Importantly, PGE2 secretion was evident in all non-induced cohorts with CD146POS spheroids showing significantly (*p* < 0.05) higher secretion levels (in average 151 pg/ml compared to 34.7 and 46.1 pg/ml for Crude and CD146NEG, respectively). As expected, upon TIC induction, PGE2 secretion was similarly increased in all three different cohorts but CD146POS had a higher trend of PGE2 secretion compared to others (Fig. 4d).

### Crude and CD146-selected IFP-MSC spheroids show enhanced immunomodulatory function in vitro

**Fig. 5:**
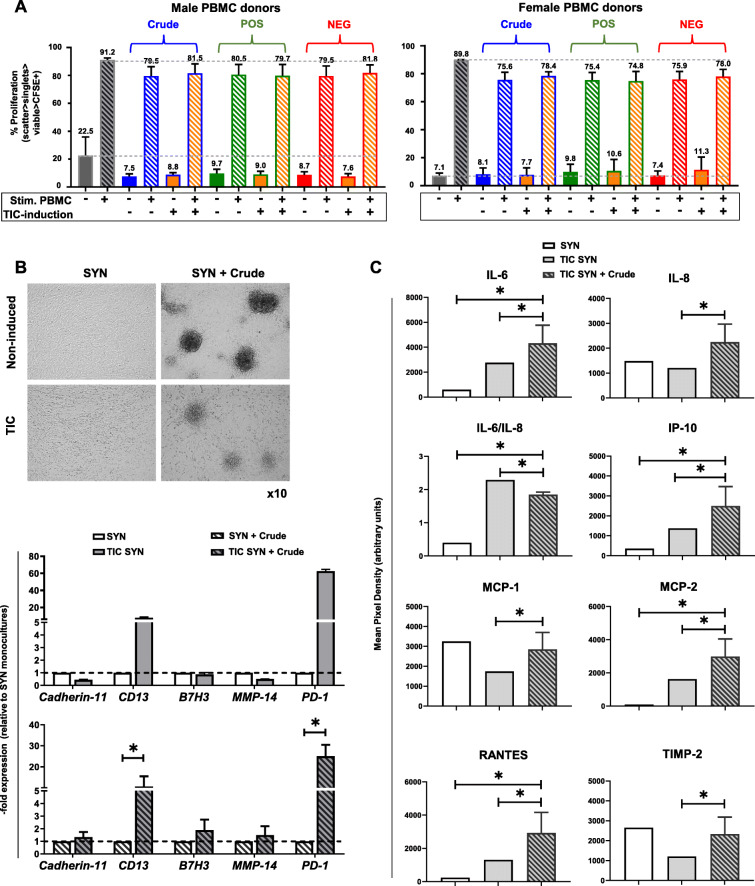
Immunomodulatory functionality of Crude IFP-MSC spheroids. **a** CFSE-labeled, PMA/IO-activated human PBMC showed a proliferation of ~ 91.2% for male PBMC donors and of ~ 89.9% for female PBMC donors, which was suppressed in a PBMC gender-dependent manner by co-culture with non-induced Crude or CD146-selected IFP-MSC spheroids (*p < 0.05, *n* = 3). **b** In both non-induced and TIC-induced SYN, non-induced Crude IFP-MSC spheroids can effectively form explant-cultures on synoviocyte monolayer whereas TIC induction of SYN significantly (*p < 0.05) upregulates inflammatory genes such as CD13 and PD-1. **c** SYN/IFP-MSC spheroid co-cultures resulted in significantly (*p < 0.05) higher secretion of immunomodulatory proteins (IL-6, IL-8, IP-10, MCP-1, MCP2, RANTES, TIMP-2) compared to TIC-induced SYN alone. All experiments were performed independently (*n* = 3)

CFSE-labeled, PMA/IO-activated human PBMC showed a proliferation of ~ 91.2% for male PBMC donors and of ~ 89.9% for female PBMC donors, which was suppressed in a PBMC gender-dependent manner by non-contact co-culture with non-induced Crude or CD146-selected IFP-MSC spheroids (Fig. 5a). On this basis, slightly enhanced PBMC suppression was observed when IFP-MSC spheroids were co-cultured with female PBMC donors. All non-induced Crude, CD146POS, and CD146NEG IFP-MSC cohorts show similar suppression capacity of the activated PBMC proliferation, ranging between 19.5 to 20.5% for male PBMC and 24.1 to 24.6% for female PBMC donors. Importantly, TIC induction of Crude or CD146-selected cohorts did not result in enhanced suppression capacity over activated PBMC.

### Effects of IFP-MSC on synoviocytes’ response to inflammation in vitro

Synoviocytes were expanded in 2D cultures until P3 showing high (> 95%) viability. To assess the capacity of IFP-MSC spheroids to attach to P3 synoviocyte monolayer in vitro and exert immunomodulatory effects, SYN/IFP-MSC spheroid co-cultures have been performed. In both non-induced and TIC-induced SYN, Crude IFP-MSC spheroids can effectively attach and form explant-cultures by expanding on a synoviocyte monolayer (Fig. 5b). Importantly, TIC induction of SYN significantly (*p* < 0.05) upregulated inflammatory genes such as CD13 and PD-1 even at the presence of IFP-MSC spheroids in culture. However, SYN/IFP-MSC spheroid co-cultures resulted in significantly (*p* < 0.05) higher secretion of 6 immunomodulatory proteins compared to TIC-induced SYN alone. Of note, SYN/IFP-MSC spheroid co-cultures result in significantly lower IL-6/IL-8 ratio from TIC-induced SYN alone, indicating the reduced inflammatory environment of such in vitro co-culture (Fig. 5c).

### IFP-MSC spheroids strongly reverse synovitis and IFP fibrosis and degrade SP in vivo

**Fig. 6:**
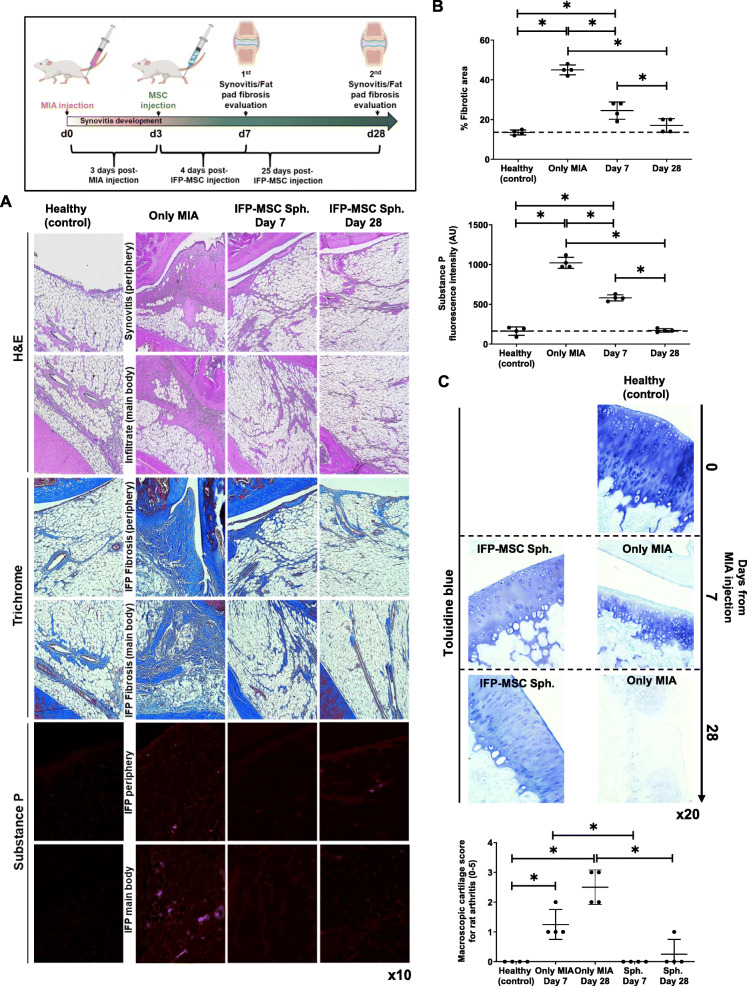
IFP-MSC spheroids effectively reverse synovitis and IFP fibrosis in vivo*.* Schematic indicating the generation of acute synovitis/IFP fibrosis rat model, therapeutic intervention, and chronological evaluation. **a**, **b** Hematoxylin/eosin staining (top 2 panels), Masson’s trichrome staining (middle two panels), and substance P immunolocalization (lower 2 panels) in sagitally sectioned knees of representative rats for healthy control, injected only with MIA or with both MIA and IFP-MSC spheroids. Compared with only MIA injected group, which showed a significant synovitis and IFP fibrosis with cellular infiltrates, a striking correlation was found between IFP-MSC spheroid treatment and the effect reducing inflammation and fibrosis after 3 and 25 days of a single intra-articular IFP-MSC injection (*p < 0.05). **c** After MIA injection, all animals developed OA changes that resulted in OA lesions on day 28. IFP-MSC spheroid injection group did not show any major OA lesion development whereas articular cartilage sGAG content was preserved which was evident by the strong toluidine blue staining (*p < 0.05)

A rat model of induced acute synovitis and IFP fibrosis was used to test the capacity of IFP-MSC spheroids to reverse synovium and IFP inflammation and fibrosis (Fig. 6). Compared to healthy rat knees, MIA group showed not only strong synovitis and fibrosis of the IFP tissue but also hyperinnervation by SP-positive sensory fibers 7 days after the intra-articular injection of MIA. Previously, we clearly demonstrated the transient engraftment of injected single cell IFP-MSC in areas of active synovitis and IFP fibrosis [28]. Herein, 4 days after IFP-MSC spheroid injection, we observed significant reduction of synovitis and IFP fibrosis (from 45 *±* 2.5 to 24.5 *±* 4.3%) that was further reversed up to day 28 (17 *±* 3.5%). According to Udo et al.’s infrapatellar fat pad inflammation scoring (0–5) for rat arthritis induced by MIA [36], only MIA (untreated) cohort developed grade 3 synovitis and grade 2 to 3 fibrosis at the main IFP body, which were independent from rat gender. In addition, SP presence was significantly diminished (1.75-fold on day 4 and 5.9-fold on day 25) after IFP-MSC injection in both peripheral areas of the IFP (close to the synovium) and inner parts (IFP body) (Fig. 6b). After 28 days of MIA injection, all animals developed articular cartilage structural changes compatible with OA lesions, mostly associated with reduction of the matrix staining and integrity. IFP-MSC spheroid injection showed articular cartilage with preserved matrix, evident by the strong toluidine blue staining of the structural sGAG. Quantification according to Udo et al.’s macroscopic scoring (0–5) for rat arthritis induced by MIA [36], only MIA (untreated) cohort developed grade 3 erosion (≤ 50% of joint surface) whereas IFP-MSC spheroid cohort showed almost intact articular surface (grade 0), which were independent from rat gender.

## Discussion

We have previously underlined the importance of ex vivo MSC “functionalization” for improved therapeutics in vivo [24]. Consequently, herein we investigated the cellular and molecular effects of spheroid culturing, CD146-based subset selection, and response to inflammatory and fibrotic environments on IFP-MSC immunomodulatory and therapeutic properties in the context of synovitis/fibrosis reversal in vivo. According to these findings, 3D spheroids confer IFP-MSC a reproducible and enhanced immunomodulatory effect in vitro and in vivo.

Importantly, our proposed gas-permeable cultures resulted in IFP-MSC spheroids without necrotic core, circumventing any oxygen and nutrients core exchange limitations associated with other spheroid culturing platforms [37]. Overall, we found that IFP-MSC spheroid culturing resulted in enhanced phenotypic, transcriptional, and secretory profiles compared to 2D cultures. This includes enrichment for “stemness” gene cohort as previously reported with other MSC types [38]; increased CD146, CD10, NG2, and CXCR4 levels; higher tripotential differentiation capacity; and significantly enhanced reparative growth factor secretion (1.9-fold and 1.32-fold, respectively). Collectively, these data demonstrate the reinforcing effect of spheroid culturing on IFP-MSC propensities even in non-induced cultures without any exogenous pro-inflammatory/pro-fibrotic priming. Upon CD146-based selection, the generated subpopulations showed distinct protein, molecular, and secretory profiles, similar to our previous report with BM-MSC [31]. As highlighted in our previous reports, CD146 and CD10 are both key molecules correlated with increased immunomodulatory MSC functionality [28–30]. However, solely 3D culture settings had significant impact on IFP-MSC subpopulation phenotype as at the transcript level *CD146* gene expression was equally upregulated between the different cohorts (Crude, CD146POS, CD146NEG) compared to 2D cultures. This finding is of outmost importance indicating that MSC manufacturing in 3D settings can boost their immunomodulatory functionality without any further induction. Furthermore, both Crude and CD146POS cohorts share 26 highly upregulated genes that are mainly involved (13 out of 26) in MSC-related/angiogenesis phenotype/function-related grouping. Taken together, these data indicate that CD146POS cohorts show similarities at the protein and transcriptional profile with Crude cohort when cultured in 3D settings in vitro.

These phenotypic and molecular differences were also reflected in the immunomodulatory secretory profile upon exposure to inflammatory (TI) or inflammatory/fibrotic (TIC) cues. The overall “signature” response to inflammation in all cohorts involves the upregulation of key immunomodulatory molecules including the T leukocyte recruitment chemokines MCP-2 and RANTES, and the immunosuppressive proteins IP-10 and MIP-1-α, that are directly correlated with the magnitude and cytokine polarity of T cell response in vivo [39–41]. In contrast, TIC inflammatory/fibrotic exposure diminished all common secreted molecules resulting in totally distinct secretory phenotypes for the three different cohorts. In terms of biological processes, various categories were highly affected that empower the IFP-MSC spheroids to respond to inflammation/injury by increasing their number, migrating to active sites of damage, and altering key cascades known to affect local immune responses.

The immunomodulatory IDO activity and PGE2 secretion were strongly induced by exposure to inflammatory/fibrotic cues as previously reported [23, 28, 30, 42], with the latter secreted in significantly higher levels in the CD146POS cohort. However, these differences were not functionally reflected in immunopotency with IFP-MSC spheroid/PBMC co-cultures. All groups showed a similar gender-independent capacity to abrogate activated PBMC proliferation in vitro, indicating that at least via soluble factors CD146-based IFP-MSC selection is not affecting the immunomodulatory functionality of IFP-MSC spheroids. Nevertheless, the comparable PBMC proliferation abrogation observed with Crude, CD146POS, and CD146NEG cohorts suggested to us that the 3D environment could be sufficient to induce and/or enhance the immunomodulatory phenotype of IFP-MSC. On the other hand, IFP-MSC spheroids could effectively induce an “immunomodulatory environment” when co-cultured with synoviocytes exposed to synovitis-mimicking TIC inflammatory/fibrotic cues, similar to a previous report with adipose-derived MSC and OA synoviocytes [43]. IFP-MSC spheroids counterbalanced the synoviocyte-induced inflammatory response via secretion of immunomodulatory molecules IP-10, MCP-1, MCP-2, RANTES, low IL-6/IL-8 ratio as well as the articular cartilage degradation inhibitor TIMP-2.

Due to the comparable immunomodulatory functionality of Crude, CD146POS, and CD146NEG cohorts in in vitro settings, we evaluated the therapeutic properties of Crude IFP-MSC spheroids in vivo. Moreover, IFP-MSC spheroid immunoevasive properties were evident as we did not observe clinical or histological signs of xeno-rejection to the human material. Similar to our previous reports using IFP-MSC in single-cell format [28, 29], we confirmed that intra-articularly injected spheroids possess strong immunomodulatory/anti-fibrotic capacity resulting in total resolution of synovitis and IFP fibrosis on day 28, as well as degradation of SP. SP is a key modulator of local inflammatory and fibrotic responses as it is involved in the modulation of immune cell proliferation, activation, and migration to sites of inflammation, and the expression of recruiting chemokines and adhesion molecules [44, 45]. Herein, the reduction or absence of SP^+^ fibers in areas of active inflammation and fibrosis for both the synovium and the body of the IFP even at day 7 indicates the strong anti-inflammatory and analgesic IFP-MSC properties in vivo. According to our previous findings [28, 29], SP degradation both in vitro and in vivo is driven by a CD10 secretion-dependent degradative mechanism that is apparently strongly effective in IFP-MSC spheroids given their stable and high CD expression. These outcomes are important as SP-secreting sensory nerve fibers predominate over sympathetic ones in anterior knee pain [46–48], whereas SP levels are increased in synovial fluid during joint inflammation [44]. Importantly, Udo et al. showed that intra-articular injection of 1.0 mg MIA in the rat knee leads early to synovitis and IFP fibrosis, and later (on day 28) to severe cartilage erosion [36]. In our study, we confirmed that IFP-MSC intra-articular injected not only result in anti-inflammatory and anti-fibrotic effects but also show strong articular cartilage protective effects.

## Conclusions

In summary, IFP-MSC 3D spheroids exhibit enhanced immunomodulatory phenotypic and transcriptional profiles, reparative growth factor secretion, and anti-inflammatory/anti-fibrotic functional cell signature both in vitro and in vivo, compared with 2D cultures. These therapeutic properties, when manufactured under minimal-manipulation conditions, facilitate the translation of proof-of-concept data into potential clinical protocols in the treatment of joint disease including synovitis, fat pad fibrosis, articular cartilage degradation, and potentially OA.

## Supplementary Information

**Additional file 1: Figure S1.** Molecular evaluation of IFP-MSC. (A) CD146 protein expression levels were significantly (*p<0.05) higher in CD146POS compared to Crude and CD146NEG 2D cultures. *CD146* gene expression levels show not only high up-regulation in 3D settings compared to 2D cultures but also higher expression in CD146NEG IFP-MSC spheroids. (B) Upon TI- or TIC- induction most of the immunomodulatory-related genes tested show up-regulated expression levels in CD146POS spheroids. All experiments were performed independently (*n*=3).

**Additional file 2: Figure S2.** Tables show the number of proteins significantly different between the three different cohorts (Crude, CD146POS, CD146NEG) upon TI or TIC induction compared to non-induced cultures.

**Additional file 3: Figure S3.** Interactome of proteins significantly up-regulated in TI or TIC conditions. All secreted proteins appeared interconnected at least through one association (except PIGF when present) and the K-means clustering networks demonstrated elevated protein-protein interaction (PPI) enrichment (*p*-value <1.0e-16) and an average local clustering coefficient >0.7 indicating that the proteins used are at least partially biologically connected. (A) In TI-induced IFP-MSC spheroids all subpopulation cohorts revealed overall similar biological processes. Crude TI-induced cohort showed higher involvement in all tested biological processes except positive regulation of cell population proliferation and angiogenesis, which showed significantly higher involvement in TI-induced CD146POS cohort (5.5% and 16.5% higher respectively) (Supplementary Figure 2A, left radar graph). (B) In TIC-induced IFP-MSC spheroids, Crude cohort showed higher involvement in 4 out of 7 biological processes tested. Compared to TI induction, TIC induction significantly decreased the angiogenesis biological process involvement in crude cohort (from 14 to 0%) and significantly increased its involvement in CD146POS cohort (from 30.7 to 41.7%) (A and B) Overall, in both TI- and TIC-induced IFP-MSC spheroids, CD146POS cohort showed significantly stronger protein involvement in 4 out of 8 KEGG reactome pathways (MAPK, PI3K-Akt, Ras, and Rap1 signaling pathways) compared to other cohorts.

**Additional file 4: Table S1.** Transcripts and primers.

**Additional file 5: Table S2.** Human mesenchymal stem cell qPCR array plate genes and their classification.

## Data Availability

All data discussed in the paper will be made available to readers upon request.

## Abbreviations

MSC: Mesenchymal stem cells
IFP: Infrapatellar fat pad
OA: Osteoarthritis
2D: Two dimensional
3D: Three dimensional
TI: Tumor necrosis factor-alpha (TNF-α) and interferon-gamma (IFN-γ)
TIC: Tumor necrosis factor-alpha (TNF-α), interferon-gamma (IFN-γ), and connective tissue growth factor (CTGF)
POS: CD146 positive
NEG: CD146 negative
PBMC: Peripheral blood mononuclear cells
PMA/IO: Phorbol 12-myristate 13-acetate and ionomycin
SYN: Synoviocytes
IDO: Indoleamine 2,3-dioxygenase
PGE2: Prostaglandin E_2_
IPA: Immunopotency assay
MIA: Mono-iodoacetate
