# Correction to: Infrapatellar fat pad-derived mesenchymal stem cell-based spheroids enhance their therapeutic efficacy to reverse synovitis and fat pad fibrosis

**DOI:** 10.1186/s13287-021-02294-w

**Published:** 2021-05-11

**Authors:** Dimitrios Kouroupis, Melissa A. Willman, Thomas M. Best, Lee D. Kaplan, Diego Correa

**Affiliations:** 1 Department of Orthopedics, UHealth Sports Medicine Institute, Miller School of Medicine, University of Miami, 1450 NW 10th Ave (3014), Miami, FL 33136 USA; 2 Diabetes Research Institute & Cell Transplantation Center, Miller School of Medicine, University of Miami, 1450 NW 10th Ave (3014), Miami, FL 33136 USA

**Correction to: Stem Cell Res Ther 12, 44 (2021)**

**https://doi.org/10.1186/s13287-020-02107-6**

The original article [1] contained typesetting errors in Figs. 2 and 4, mistakenly introduced by the production team that managed this article.

These errors have both since been corrected.
