# Supplementary material for: Infrapatellar fat pad-derived mesenchymal stem cell-based spheroids enhance their therapeutic efficacy to reverse synovitis and fat pad fibrosis
Source: Stem Cell Res Ther. 2021 Jan 7;12:44. doi: 10.1186/s13287-020-02107-6 (PMC7792122; doi:10.1186/s13287-020-02107-6)
Supplement: Supplementary file 4 — Additional file 4: Table S1. Transcripts and primers. [file 13287_2020_2107_MOESM4_ESM.docx]

| **TRANSCRIPT NAME** | **PRIMERS** |
| --- | --- |
| *BGLAP* | Forward: *CAG CGA GGT AGT GAA GAG AC*  Reverse: *TGA AAG CCG ATG TGG TCA G* |
| *OMD* | Forward: *ACG ATG ATC CTG ACA ATG CTC*  Reverse: *GTA TAG GTT TTG TGA AGT CGT AAG TG* |
| *FABP4* | Forward: *AAG AAG TAG GAG TGG GCT TTG*  Reverse: *TCA ACG TCC CTT GGC TTA TG* |
| *PPARγ* | Forward: *GCC TGC ATC TCC ACC TTA TT*  Reverse: *AGC GGG AAG GAC TTT ATG TAT G* |
| *ACAN* | Forward: TGT GGG ACT GAA GTT CTT GG  Reverse: *AGC GAG TTG TCA TGG TCT G* |
| *COMP* | Forward: GAC AGT GAT GGC GAT GGT ATA G  Reverse: *TCA CAA GCA TCT CCC ACA AA* |
| *IL-6* | Forward: *GTA GCC GCC CCA CAC AGA CAG CC*  Reverse: *GCC ATC TTT GGA AGG TTC* |
| *IL-8* | Forward: *GAA CTG AGA GTG ATT GAG AGT*  Reverse: *CTT CTC CAC AAC CCT CTG* |
| *CD10* | Forward: *CTG TGG GAT GAG GAG GTT AAA G*  Reverse: *GGA GGC TAA AGC AGG AGA ATA G* |
| *ICAM-1* | Forward: AGG AGG TGG TAA GAG AGA AGA G  Reverse: *TAA GGG TGG GAG GAG GAT TT* |
| *G-CSF* | Forward: AGC TTC CTG CTC AAG TGC  Reverse: *TTC TTC CAT CTG CTG CCA GAT GGT* |
| *HGF* | Forward: ATG TGG GTG ACC AAA CTC CTG  Reverse: *CTA TTG AAG GGG AAC CAG AGG* |
| *IL-10* | Forward: GCC TAA CAT GCT TCG AGA TC  Reverse: *TGA TGT CTG GGT CTT GGT TC* |
| *HLA-G* | Forward: GCT GCT GTG CTG TGG AGA A  Reverse: *TCT GGA ACA GGA AAG GTG ATT GG* |
| *IDO* | Forward: *AGA GTC AAA TCC CTC AGT CC*  Reverse: *AAA TCA GTG CCT CCA GTT CC* |
| *GAPDH* | Forward: TAC GTC GTG GAG TCC ACT GG  Reverse: *GCC AAC GTG TCA GTG GTG GA* |

**Supplementary Table S1.** Transcripts and primers.
