# Supplementary material for: Infrapatellar fat pad-derived mesenchymal stem cell-based spheroids enhance their therapeutic efficacy to reverse synovitis and fat pad fibrosis
Source: Stem Cell Res Ther. 2021 Jan 7;12:44. doi: 10.1186/s13287-020-02107-6 (PMC7792122; doi:10.1186/s13287-020-02107-6)
Supplement: Supplementary file 5 — Additional file 5: Table S2. Human mesenchymal stem cell qPCR array plate genes and their classification. [file 13287_2020_2107_MOESM5_ESM.docx]

**Supplementary Table S2.** Human mesenchymal stem cell qPCR array plate genes and their classification.

| **TRANSCRIPT NAME** | **CLASSIFICATION** |
| --- | --- |
| *PPARG, RHOA, CEBPA, CEBPB, LEPR* | Adipogenic |
| *HAT1, ITGAX, KAT2B, SOX9, ACAN, COL2A1, MMP13, CSPG4* | Chondrogenic |
| *BMP4, TGFB1, TGFB3, BMP6, KDR, MSX1, MSX2, BMP2, PRRX1* | Chondrogenic / Osteogenic |
| *TWIST2, ANPEP, CASP3, CD44, ENG, ERBB2, FUT4, FZD9, ITGA6, ITGAV, MCAM, NGFR, NT5E, PDGFRB, PROM1, THY1, VCAM1, BDNF, CD200, COL10A1, FGF18, DLX2, DLX5, PDGFRA* | MSC |
| *CTNNB1, EGF, HGF, ICAM1, IFNG, IGF1, IL10, IL1B, IL6, ITGB1, KITLG, MMP2, NES, NUDT6, PTPRC, SLC17A5, TNF, VEGFA, VIM, VWF, JAG1, NOTCH1, GDF15, SMAD4, HIC1* | MSC-related/Angiogenesis |
| *ALPL, IBSP, SP7, BGLAP, BMP7, COL1A1, FGF10, HDAC1, PTK2, SMURF1, SMURF2, TBX5, RUNX2, FGF9* | Osteogenic |
| *FGF2, LIF, SOX2, TERT, GDF5* | Stemness |
| *GAPDH, RN18S1, ACTB, TBP, UBC*  Positive Control | Housekeeping / Control |
