# Supplementary material for: Infrapatellar fat pad-derived mesenchymal stem cell-based spheroids enhance their therapeutic efficacy to reverse synovitis and fat pad fibrosis
Source: Stem Cell Res Ther. 2021 Jan 7;12:44. doi: 10.1186/s13287-020-02107-6 (PMC7792122; doi:10.1186/s13287-020-02107-6)
Supplement: Supplementary file 3 [file 13287_2020_2107_MOESM1_ESM.pdf]

## Slide 1

Supplementary Figure 1

Kouroupis et al

2D cultures
Spheroids

CD146

CD146

CD146

A

B

TI - priming

TIC - priming

CRUDE

CD146POS

-fold expression
 (relative to non-induced cultures)

-fold gene expression

% CD146 expression 

CD146 protein

CD146 transcript

17±17%

89±4%

12±3%
