# Supplementary material for: Infrapatellar fat pad-derived mesenchymal stem cell-based spheroids enhance their therapeutic efficacy to reverse synovitis and fat pad fibrosis
Source: Stem Cell Res Ther. 2021 Jan 7;12:44. doi: 10.1186/s13287-020-02107-6 (PMC7792122; doi:10.1186/s13287-020-02107-6)
Supplement: Supplementary file 4 [file 13287_2020_2107_MOESM2_ESM.pdf]

## Slide 1

A

Kouroupis et al

B

Supplementary Figure 2

Fractions
Significantly (p<0.05)
 UP-regulated 
post-TI stimulation
N
Significantly (p<0.05)
 DOWN-regulated 
post-TI stimulation
N
Crude
IFN-γ, IL-6, IL6 sR, IL-8, IP-10, MCP-2, M-CSF, MIP-1-α, MIP-1-β, RANTES, TNF-α, EGF R, NT-3, TGF-β2
14
Eotaxin-2 , G-CSF, GM-CSF, sTNF-RI, PDGF-BB, FGF-2, β-NGF, EGF, FGF-4, GDNF, HGF, IGFBP-1, M-CSF R
13
POS
ICAM-1, IFN-γ, IL-4, IL-6, IP-10, MCP-2, MIP-1-α, RANTES, TNF-α, AR, FGF-2, β-NGF, EGF, EGF R, GM-CSF, IGFBP-1, M-CSF R, NT-4, PDGFR-α, PDGF-AB, TGF-β1, TGF-β2, VEGF-A, VEGF R2, VEGF R3, VEGF D
26
SCF
1
NEG
ICAM-1, IFN-γ, IL-8, IP-10, MCP-2, MIP-1-α, RANTES, TNF-α, PIGF
9
IL-15, IL-16, TNF-β, sTNF-RI, β-NGF, FGF-4, IGFBP-1, IGFBP-3
VEGF R2
9

Fractions
Significantly (p<0.05)
 UP-regulated 
post-TIC stimulation
N
Significantly (p<0.05)
 DOWN-regulated 
post-TIC stimulation
N
Crude
ICAM-1, IFN-γ, IL-8, IL-12-p40 (IL-12-β), IL-12-p70 (IL-12-β),  IL-15, IP-10, MCP-2, M-CSF, MIP-1-α, MIP-1-β, MIP-1-δ, RANTES, TNF-α, NT-3, TGF-α, TGF-β1, TGF-β2
18
sTNF-RI, PDGF-BB, FGF-2, EGF, FGF-4, IGF-I, IGF-I sR, PDGF-AA, PDGF-AB, PIGF, SCF
11
POS
ICAM-1, IFN-γ, IP-10, MCP-2, MIP-1-α, RANTES, TNF-α, AR, FGF-2, EGF, FGF-6, IGFBP-1, IGF-I, PDGFR-α, PDGFR-β, PDGF-AA, PDGF-AB, PIGF, SCF R, TGF-β2, TGF-β3, VEGF-A, VEGF R2, VEGF R3
24
Eotaxin-2
1
NEG
G-CSF, IL-3, IL-16, IGFBP-1, IGFBP-3
5
ICAM-1, IFN-γ, IL-8, IL-12-p70 (IL-12-β), IP-10, MCP-2, MIP-1-α, MIP-1-δ, RANTES, TNF-α, sTNF-RII, HB-EGF
12
