# Supplementary material for: Infrapatellar fat pad-derived mesenchymal stem cell-based spheroids enhance their therapeutic efficacy to reverse synovitis and fat pad fibrosis
Source: Stem Cell Res Ther. 2021 Jan 7;12:44. doi: 10.1186/s13287-020-02107-6 (PMC7792122; doi:10.1186/s13287-020-02107-6)
Supplement: Supplementary file 5 [file 13287_2020_2107_MOESM3_ESM.pdf]

## Slide 1

Supplementary Figure 3

Kouroupis et al

A

UP-regulated TI-induced IFP-MSC

Crude

POS

NEG

UP-regulated TIC-induced IFP-MSC

Crude

POS

NEG

B

Biological Processes

KEGG reactome

Biological Processes

KEGG reactome

Crude

POS

NEG

Crude

POS

NEG
